# A Molecular Function Map of Ewing's Sarcoma

**DOI:** 10.1371/journal.pone.0005415

**Published:** 2009-04-30

**Authors:** Maximilian Kauer, Jozef Ban, Reinhard Kofler, Bob Walker, Sean Davis, Paul Meltzer, Heinrich Kovar

**Affiliations:** 1 Children's Cancer Research Institute, St. Anna Kinderkrebsforschung, Vienna, Austria; 2 Biocenter, Division of Molecular Pathophysiology, Medical University Innsbruck, Innsbruck, Austria; 3 Genetics Branch, National Cancer Institute, National Institutes of Health, Bethesda, Maryland, United States of America; Institute of Cancer Research, United Kingdom

## Abstract

**Background:**

EWS-FLI1 is a chimeric ETS transcription factor that is, due to a chromosomal rearrangement, specifically expressed in Ewing's sarcoma family tumors (ESFT) and is thought to initiate the development of the disease. Previous genomic profiling experiments have identified EWS-FLI1–regulated genes and genes that discriminate ESFT from other sarcomas, but so far a comprehensive analysis of EWS-FLI1–dependent molecular functions characterizing this aggressive cancer is lacking.

**Methodology/Principal Findings:**

In this study, a molecular function map of ESFT was constructed based on an integrative analysis of gene expression profiling experiments following EWS-FLI1 knockdown in a panel of five ESFT cell lines, and on gene expression data from the same platform of 59 primary ESFT. Out of 80 normal tissues tested, mesenchymal progenitor cells (MPC) were found to fit the hypothesis that EWS-FLI1 is the driving transcriptional force in ESFT best and were therefore used as the reference tissue for the construction of the molecular function map. The interrelations of molecular pathways were visualized by measuring the similarity among annotated gene functions by gene sharing. The molecular function map highlighted distinct clusters of activities for EWS-FLI1 regulated genes in ESFT and revealed a striking difference between EWS-FLI1 up- and down-regulated genes: EWS-FLI1 induced genes mainly belong to cell cycle regulation, proliferation, and response to DNA damage, while repressed genes were associated with differentiation and cell communication.

**Conclusions/Significance:**

This study revealed that EWS-FLI1 combines by distinct molecular mechanisms two important functions of cellular transformation in one protein, growth promotion and differentiation blockage. By taking MPC as a reference tissue, a significant EWS-FLI1 signature was discovered in ESFT that only partially overlapped with previously published EWS-FLI1–dependent gene expression patterns, identifying a series of novel targets for the chimeric protein in ESFT. Our results may guide target selection for future ESFT specific therapies.

## Introduction

Ewing's sarcoma family tumors (ESFT), which comprise Ewing's sarcoma, peripheral primitive neuroectodermal tumors, and Askin tumor, are undifferentiated small blue round cell tumors affecting children and young adults as the second most frequent bone cancer [1]. This highly aggressive cancer is characterized by a chromosomal translocation that results in the formation of a gene fusion between the *EWSR1* locus and an ETS transcription factor gene, which in 85% of the cases is *FLI1* [2]. *EWS-ETS* fusion genes encode aberrant transcription factors which are thought to be rate-limiting for ESFT pathogenesis [3]. Using different model systems the functional consequences on gene expression of EWS-FLI1 have recently been studied in whole genome gene expression profiling analyses, and the target gene sets were compared to deregulated genes in ESFT to test for biological relevance (see [4] for a review). In this study we follow this approach, but while previous reports mostly focused on the functional relevance of single selected target genes, we aimed at analyzing the molecular function of EWS-FLI1 regulated genes in ESFT on a pan-genomic level. Therefore we highlighted classes of genes, rather than single genes that appear to be crucial for the development of ESFT and consequently deserve to be studied in more detail.

In the absence of knowledge about the tissue of origin for ESFT, previous studies measured EWS-FLI1 regulated gene expression relative to either other sarcomas [5], [6] or a mean of different normal tissues [6], [7]. Tirode et al. [8] recently showed that the profiles of different EWS-FLI1-silenced ESFT cell lines converge toward that of mesenchymal progenitor cells (MPC). Furthermore, it was shown that EWS-FLI1 induces a gene expression profile in human MPC that resembles that of ESFT [9] and that EWS-FLI1 can transform murine primary bone marrow-derived mesenchymal progenitor cells and induce ESFT resembling tumors in mice [10], [11]. Here, we demonstrate that using MPC as reference tissue best fits the model that EWS-FLI1 is the major driver for the gene expression signature of ESFT.

## Results

### Defining a reference tissue for ESFT

**Figure 1:**
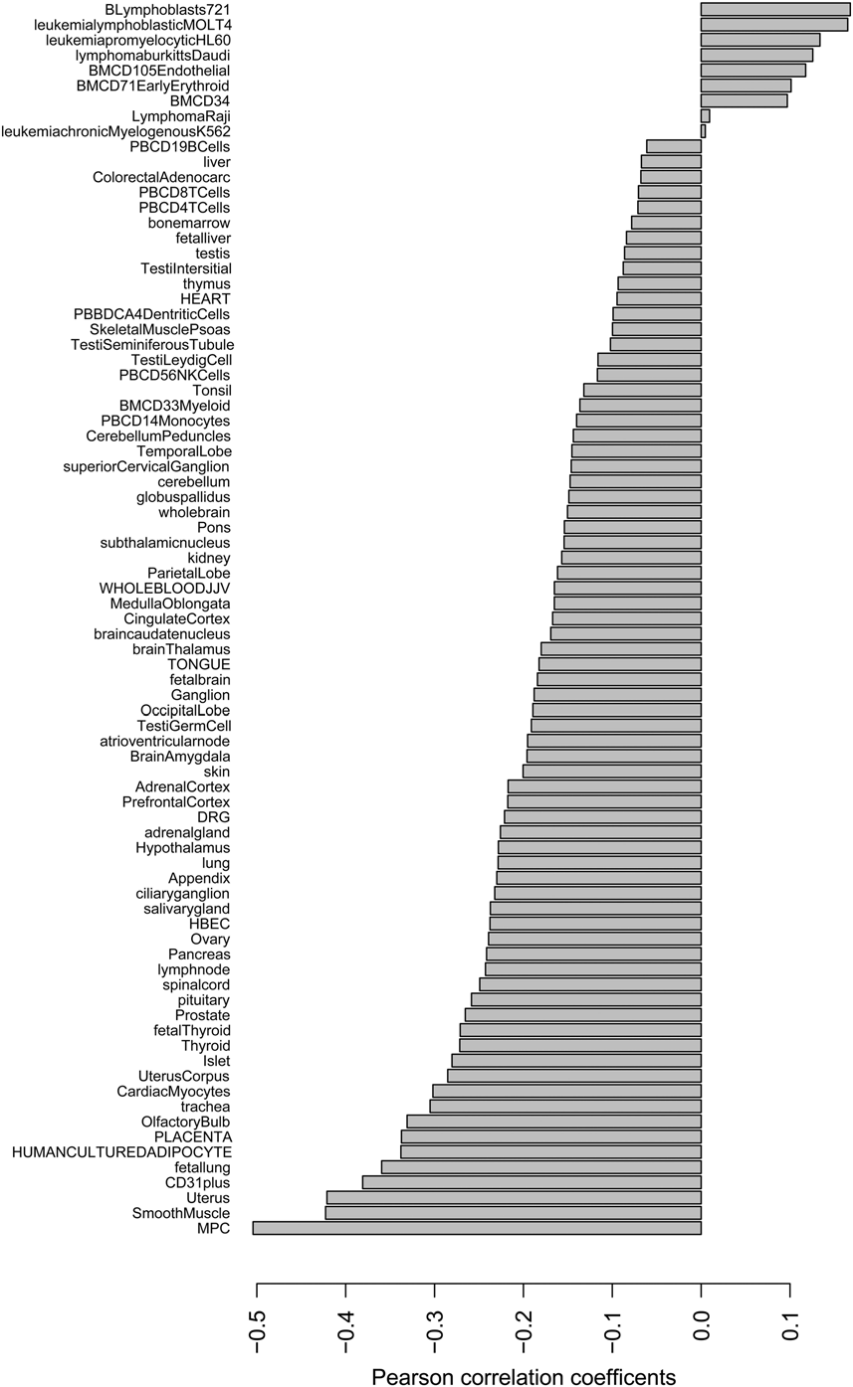
Plot of Pearson correlation coefficients of corr(ΔET, ΔKD). For each reference tissue tested (y-axis) the correlation coefficient between ΔET_[ref]_ and ΔKD is shown (x-axis). While the distribution of correlation coefficients is generally skewed towards negative values the highest negative correlation is found for corr(ΔET_[MPC]_, ΔKD).

In order to construct a molecular function map of ESFT, information about EWS-FLI1 regulated genes was combined from two sources using the same microarray platform. On the one hand consistently altered gene expression after 96 hours EWS-FLI1 knock down by fusion type specific shRNA [12] was determined in 5 ESFT cell lines (TC252, STA-ET-1, WE68, STA-ET-7.2, SK-N-MC), the largest panel of ESFT cell lines used for this purpose so far. On the other hand, genes were identified that are consistently de-regulated in a panel of 59 primary ESFT. To assess de-regulation of genes in ESFT, gene expression has to be compared to a reference tissue, the choice of which critically influences the results. However, the tissue of origin of ESFT, which would be the ideal reference, is not known. The assumption that most gene expression aberrations in ESFT with respect to the cell of origin are triggered by EWS-FLI1 provides a model with which it is possible to test the quality of any reference tissue. More specifically, gene expression differences between ESFT and the reference tissue (ΔET) are expected to be inversely correlated with the gene expression differences between EWS-FLI1 knockdown and control (ΔKD). Therefore, by maximizing this expected correlation we searched for the tissue which fits the model assumption best. To this end we compared gene expression differences between primary ESFT and all 79 tissues in the Novartis gene expression atlas [13] as well as a set of recently published MPC data from two sources [8], [14], and subsequently searched for the tissue that maximizes the inverse correlation with the gene expression change after a EWS-FLI1 knockdown. We found that, while the correlations were generally biased towards negative values (Figure 1), the highest inverse correlation was found for the comparison of ΔKD with ΔET from the comparison of ESFT with MPC as reference tissue (ΔET_[MPC]_, r = −0.5). Hence using a different analytical approach, we corroborated the findings of Tirode et al. [8] with data from more than twice as many primary ESFT and more cell line models.

**Figure 2:**
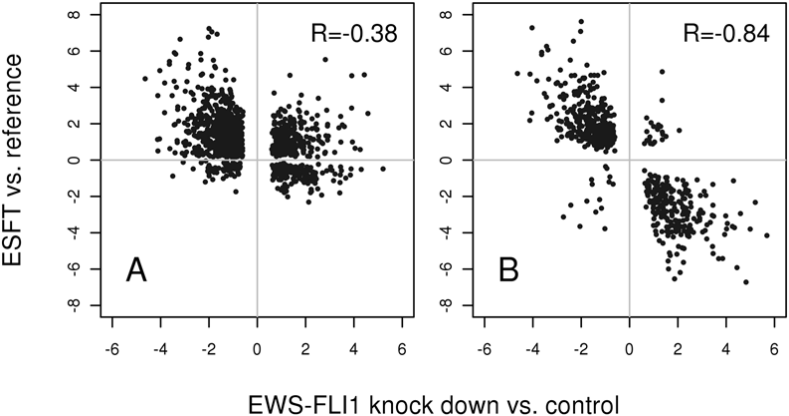
ΔKD-ΔET plot. Plot of relative gene expression values of the EWS-FLI1 knockdown compared to control (ΔKD, x-axis) and ESFT compared to reference (ΔET, y-axis). (A) reference = the mean of all tissues in the Novartis gene expression atlas, (B) reference = human mesenchymal progenitor cells (MPC). Genes for this plot were selected by applying an adjusted p-value cut-off: P<0.1 for ΔKD and ΔET. The Pearson correlation coefficients (R) between ΔKD and ΔET are indicated in the boxes.

In Figure 2 the intersection of the most significant genes (FDR<0.1, see below and Methods) from ΔET and ΔKD is plotted against each other using either the mean of all tissues (Figure 2A) or MPC (Figure 2B) as a reference. In Figure 2A many genes do not conform to the expectation for EWS-FLI1 regulated genes since they are repressed by EWS-FLI1 but expressed at a higher level in the tumors than in the reference (upper right quadrant). In contrast, almost all genes conform to the hypothesis in Figure 2B. Here, the inverse correlation with knockdown data improves from −0.38 for the ESFT-mean tissue comparison to −0.84 for the ESFT-MPC comparison. The increased correlation fits the model of comparing ESFT gene expression to the appropriate base level of the presumptive tissue of origin. Therefore, for all further analyses we chose MPC as gene expression reference for ESFT.

### Defining EWS-FLI1 target genes

Genes consistently regulated by EWS-FLI1 in cell line models and primary tumors were identified by applying thresholds of different stringencies to the two gene lists (adjusted P-value cutoffs: 0.1 and 0.25, gene sets “GS1” and “GS2”, respectively. For details, see Materials and Methods section). Furthermore, a small fraction of genes that did not follow the inverse correlation between ΔKD and ΔET by falling into the upper right and lower left quadrants of Figure 2B were excluded. The percentage of such genes corresponded to ∼6% in GS1 and ∼12% in GS2. In the framework of our starting hypothesis these genes likely represent false positives and therefore their percentage can be used as empirical estimates of the false discovery rate in GS1 and GS2. It can, however, not be excluded that in the enigmatic tissue of ESFT origin, which according to our data should be very close to MPC, these few outlier genes may in fact behave according to the hypothesis. Applying these criteria we found 237 and 344 (GS1) genes to be consistently up- and downregulated in the EWS-FLI1 knockdown cell lines, and repressed and activated in primary tumors at a FDR∼<0.06. For GS2, 731 and 463 genes were identified as EWS-FLI1 up- and down-regulated (FDR∼<0.12). The complete lists comprising results for all genes from the analysis of differential expression can be found in Table S1.

### Comparison of EWS-FLI1 target genes to literature data

The gene sets GS1 and GS2 were compared to a recently published “core EWS-FLI1 gene expression signature” [4]. As a result we found that, while the data sets significantly overlap (P<10e-20, hypergeometric test), most genes in this earlier gene expression signature fall below the significance threshold (of either ΔET or ΔKD) in our data, and we identified many additional genes as EWS-FLI1 regulated even using the stringent gene set GS1. Overall 457 of the up- and 198 down regulated genes in the Hancock data set were present in our data set of 7034 genes. Out of these, 115 and 50 genes overlapped with GS1 and 181 and 75 with GS2.

The data were also compared to a recently published set of putative direct binding targets of EWS-FLI1 as determined by ChIP-on-chip [15]. Only 19 (5.5%) and 7 (2.9%) of the 903 putative direct binding targets were found in the up- and down-regulated genes of GS1. In GS2 these numbers are 28 and 16 (3.8% and 3.5%) which is similar to the overlap with the Hancock gene set: 23 and 11 genes (3.9% and 3.8%) although different genes are affected.

### A molecular function map of EWS-FLI1 target genes

A molecular function map of EWS-FLI1 target genes was constructed by annotating the stringent set of EWS-FLI1 regulated genes defined above (GS1) using data bases of gene-function relationships. Gene annotations in these data bases are highly redundant and therefore we followed a two tiered strategy: first annotations significantly enriched in the EWS-FLI1 signature were identified, and then these different functional groups were aggregated into a map by using the number of shared genes as similarity criterion (gDist, see Methods).

**Figure 3:**
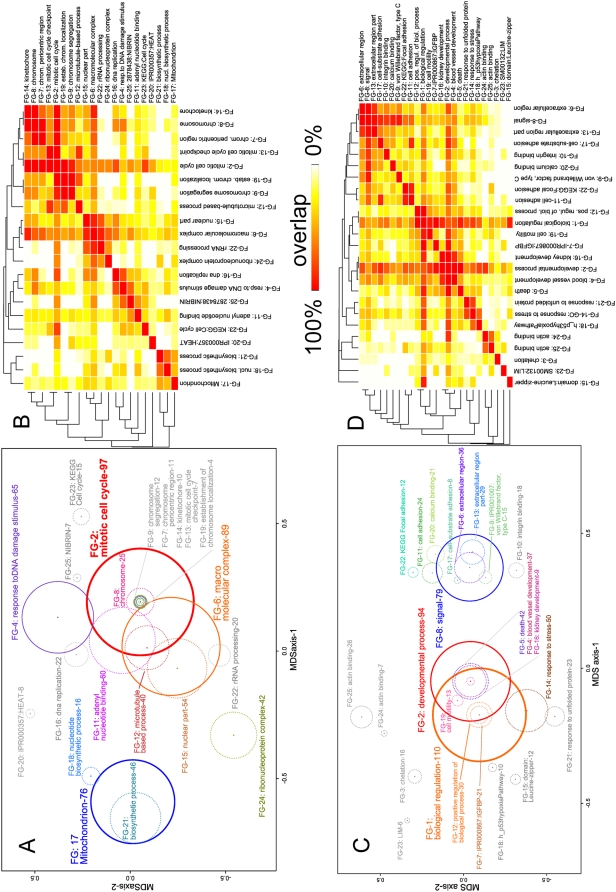
Molecular Function Map of ESFT. Functional groups (FG) from the DAVID analysis were aggregated by multidimensional scaling (MDS – A: EWS-FLI1 upregulated genes and C: EWS-FLI1 downregulated genes) and hierarchical clustering (B: EWS-FLI1 upregulated genes and D: EWS-FLI1 downregulated genes). In the MDS plots the x- and y-axis represent the first two dimensions of the MDS procedure (see Materials and Methods for details). Circles indicate the relative size (number of genes) of the annotation clusters. FGs with more than 60 genes are shown as solid circles, smaller FGs as dashed circles. The number of genes within each FG is indicated after the hyphen. The intersections of the circles are determined by their position in the plot and their size only and do not quantify the number of shared genes. However, FGs (circles) which share the same center indicate that the smaller gene set is a subset of the larger gene set. The heatmaps (B, D) show a hierarchical clustering (average linkage algorithm) of the FGs using the number of shared genes as distance measure (gDist – see Methods). Red indicates highest similarity (the smaller gene set is a subset of the larger one) and white the lowest similarity (no shared gene). Intermediate values for gDist are indicated as shades of orange/yellow.

Functional groups (FG) of gene annotations in the 344 EWS-FLI1 up- and 237 down-regulated genes of GS1 were identified using “The Database for Annotation, Visualization and Integrated Discovery” (DAVID) [16]. The full output of this analysis can be inspected in Table S2, S3. To construct a map of molecular functions, the interrelations between the 25 most significant FGs were explored by multidimensional scaling (MDS) (Figure 3A and 3C) and hierarchical clustering using gDist (Figure 3B and D). To improve the visualization of the clustering results, broadly defined general FGs consisting of more than 200 genes were omitted in this analysis (i.e. FG1, FG3 and FG10 in Table S2).

For the EWS-FLI1 up-regulated gene set (Figure 3A and 3B) the largest molecular function group is FG-2 - “mitotic cell cycle” - which comprises five more FGs that share the same center in the MDS plot. Therefore all genes in these five FGs are also annotated with a cell cycle related function. Cell cycle thus represents the major hub of molecular functions for EWS-FLI1 up-regulated genes. The central role of this functional group can also be seen in the heatmap (Figure 3B) which shows that FG-2 is the group that is most highly connected by gene-sharing (highest values of gDist) to other FGs. The second most connected FG is FG-6 - “macromolecular complex”. Only three functional groups are located very far apart from FG-2 (and FG-6) in gene space, the largest of which is FG-17 – “mitochondrion” which shares most genes with FG-18 and FG-21 (both “biosynthetic process”). Two additional prominent (sub-) clusters can be discerned in the heatmap comprising FG-6, -15, -22 and -24, and FG-11, -4, -16 and -25. FG-20 (“Interpro motif HEAT”) and FG-25 (“NIBRIN”) are small specific groups and both are only marginally significant (Table S2). FG-20 comprises 8 genes which contain the “HEAT repeat” which is found in cytoplasmic proteins that may be involved in intracellular transport processes. FG-25 consists of seven genes (MSH2, RFC4, MSH6, PRKDC, RMI1, CCND1, CCNB1) which interact with at least one of: NIBRIN (NBS2), RAD50, BLM, RFC1, MLH1, MRE11 or BRCA1. All of these seven proteins are annotated to be bound by MSH2, RFC4 and MSH6. These are the most significant protein interactions found for upregulated genes, and the most significant annotation of this group is “DNA-repair” and “response to DNA damage stimulus”. The only other significantly enriched protein-interaction term was p21 (CDKN1A) which is found in FG-23 (interacting genes from GS1: CDC7, CCNB2, CCND1, CCNB1). To address the question whether the down regulation of cell cycle related genes might result from an indirect effect of a growth arrest of cell lines after the knockdown of EWS-FLI1 we performed a DAVID analysis of significantly downregulated genes (P<0.01 after Benjamini-Hochberg correction) from the earliest (18 hours after knockdown) time point of a Doxicyclin inducible knockdown of EWS-FLI1 in A673 cells. GO:0007049 (cell cycle) was identified as highly significantly overrepresented in this gene set (P = 1.3E-10) with 72 genes annotated in this category (data not shown).

The molecular function map for the EWS-FLI1 down-regulated genes shows two main clusters. On the right in Figure 3C the main functional group is FG-8 – “signal”. This group completely comprises FG-6, “extracellular region”, and seven other FGs are located in close proximity. Gene sharing between all of these groups is very high (Figure 3D). Three of these groups contain cell adhesion related genes and one group integrin binding genes. FG-9 consists of 15 genes which either contain the VWC domain which is found in proteins that form multiprotein complexes and/or which are involved in polysaccharide binding, glycosaminoglycan binding or heparin binding. The main functions of these genes are again cell adhesion, migration, homing, pattern formation, and signal transduction. On the left side of Figure 3C, FG-1 and FG-2, which share ∼75% of the genes, represent the main functional groups. Apoptosis (FG-5 “death”) related genes, blood vessel development (FG-4) are comprised within FG-2, and FG-7 is a subset of FG-1 but shares also 95% of the genes with FG-2. FG-7 contains not only five genes with an IGFBP related protein domain (CRIM1, IGFBP3, CTGF, HTRA1, CYR61) but more generally also genes involved in cell growth and morphogenesis. FG-14 is the only other larger functional group. It contains genes with functions related to “response to stress”, “blood coagulation”, “response to wounding” and clusters with FG-21 and FG-18. In FG-18 members of the p53 (KEGG) and p53-hypoxia (Biocarta) pathway are found (CDKN1A, DNAJB1, FHL2, GADD45A, GADD45B, IGFBP3, NQO1, SERPINE1 and THBS2) and also interactions of these genes among each other and with other proteins mainly of the p53 pathway: TP53, E1A, GADD45A, MAPK8, FLH2, IGFBP3, BAX, RPA1, ABCB1, TAF1. Apart from FHL2 also five other LIM-domain (a protein domain that mediates protein-protein interactions) containing genes are found in GS1 (LIMS2, LPP, CSRP2, PDLIM2, FHL2, LIMA1) which are collected in FG-23. Another protein domain that is overrepresented in the EWS-FLI1 down regulated gene set is the Leucine-zipper which is represented in FG-15 where 11 genes with transcription factor activity (FOSL2, BACH1, MAFF, ATF3, FOSL1, XBP1, CEBPD, TSC22D3, EPAS1, TCF7L1, TCF7L2) are combined, three of these from the Wnt pathway. One of the most significant molecular function groups of EWS-FLI1 repressed genes is FG-3 which combines genes involved in chelation/metal ion binding and acetylation. Interestingly the whole Metallothionein cluster on chromosome 16q13 is found on the down regulated side of GS1.

To examine the relationship of the EWS-FLI1 deregulated gene signature with publically available data sets we searched a large database of molecular signatures (MSigDB available from the Broad Institute (http://www.broad.mit.edu/gsea/msigdb/ Cambridge, USA) for significant overlap with EWS-FLI1 deregulated genes using an approach (pGSEA) that does not require applying a P-value threshold to filter for significant genes prior to the analysis [17]–[19]. The MSigDB database consists of five different gene collections: i) C1: positional gene sets, ii) C2: curated gene sets from canonical pathways and experimental data, iii) C3: DNA motif gene sets for transcription factor and miR binding sites, iv) C4: computational gene sets and v) C5: GO terms. C1–C3 were used in this analysis. In contrast to the DAVID analysis where a pre-defined gene set was tested (GS1), the input for pGSEA was the whole filtered expression data set (7034 genes – Table S1), and ΔET and ΔKD were tested independently from each other. Despite testing ΔET and ΔKD independently we found that significantly scoring gene sets from MSigDB (P<10e-4) were almost always expressed in opposite directions in the two experimental data sets consistent with our starting hypothesis about the inverse correlation of ΔET and ΔKD (Table S4). Overall, at a stringent P value cutoff of P<10e-4, 16 MSigDB gene sets were found to be significant for c1 (3 ΔKD/ΔET down/up, 13 ΔKD/ΔET up/down), 142 for c2 (96 ΔKD/ΔET down/up, 46 ΔKD/ΔET up/down) and 55 for c3 (30 ΔKD/ΔET down/up, 25 ΔKD/ΔET up/down). Lists of all significant MSigDB datasets, the respective pGSEA statistics and the contributing genes can be found in Table S4.

**Figure 4:**
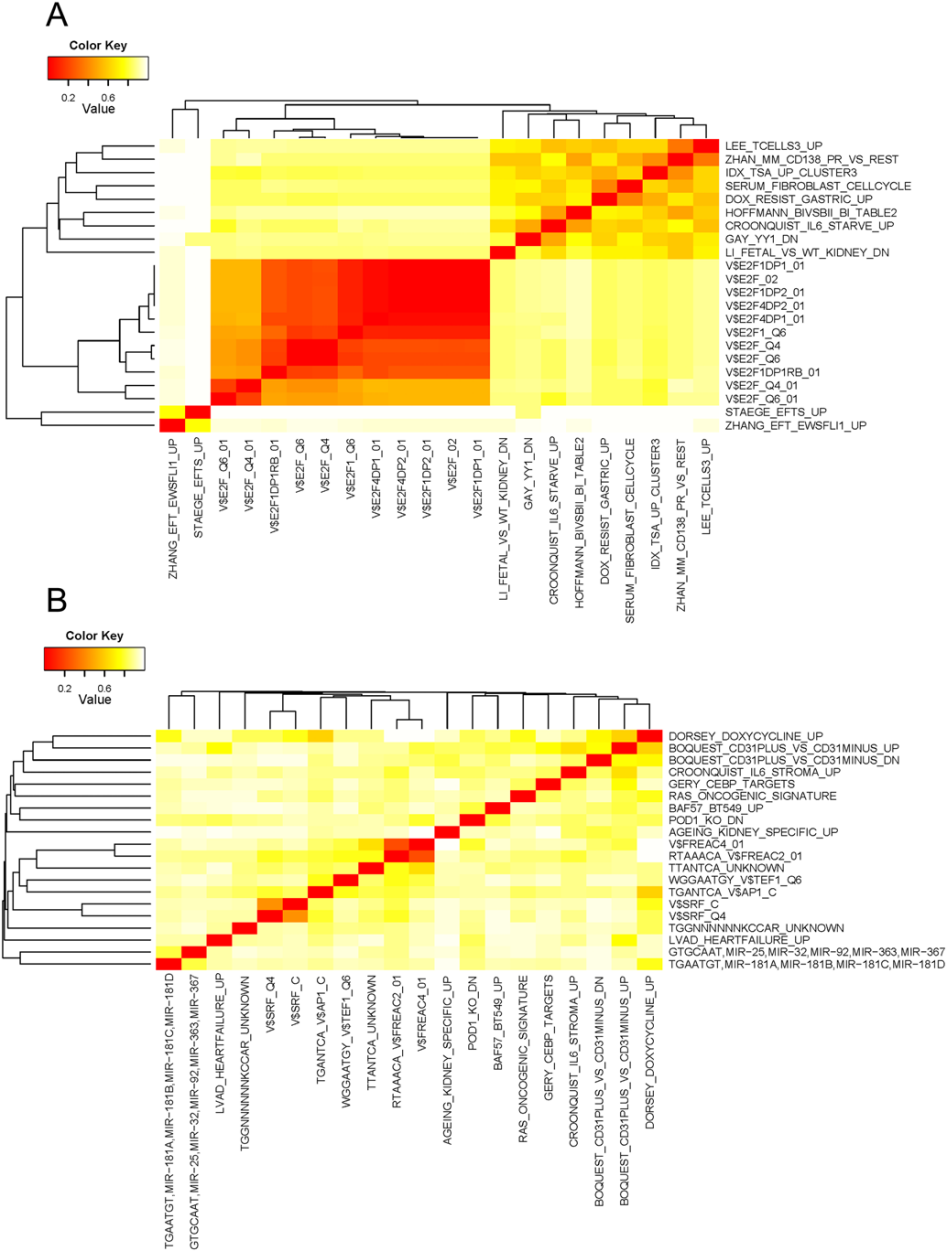
Significant MSigDB gene sets. Significant MSigDB gene sets (c2, c3) from the pGSEA analysis were aggregated by hierarchical clustering. A: Gene sets significantly overlapping EWS-FLI1 upregulated genes, B: Gene sets significantly overlapping EWS-FLI1 downregulated genes.

The similarity among the significant MSigDB gene collections was further explored by identifying the ten most significant gene sets from c2 and c3 by their combined z-score (see Methods), and their proximity in gene space was determined using gDist. The resulting distance matrix was used for hierarchical clustering of the MSigDB gene sets (Figure 4). The emerging picture for EWS-FLI1 up- versus down-regulated gene sets was very different. In the up-regulated class (Figure 4A) one large cluster could be found comprising the majority of the 20 tested MSigDB gene sets except the ones with E2F binding motifs, which formed the second cluster, and two gene sets that were found to be up-regulated in ESFT in earlier studies [20], [21], which form a separate group. For the down-regulated class on the other hand (Figure 4B) the molecular signatures from MSigDB showed very little clustering. As discussed below this finding might hint to a more complex transcription-regulatory network acting upstream of the EWS-FLI1 down-regulated than of EWS-FLI1 up-regulated genes.

## Discussion

EWS-ETS gene fusions (predominantly EWS-FLI1) are associated with at least 95% of ESFT. They sometimes occur as the only detectable genetic aberration in this disease and are therefore assumed the major driver of aberrant gene expression in ESFT [3]. Consequently, EWS-FLI1 is considered the ideal target for future ESFT treatment strategies [22]. While it is still difficult to directly target aberrant transcription factors, intervention into EWS-FLI1 driven downstream pathways may be feasible. Since it is unlikely that ESFT pathogenesis solely depends on the function of single EWS-FLI1 target genes, this study was performed to define on a pan-genomic level EWS-FLI1 driven functional pathways in ESFT. It has previously been reported that the EWS-FLI1 transcriptional target spectrum depends on the cellular context [3], [23], [24]. Thus, the identification of EWS-FLI1 regulated genes and pathways relevant to ESFT pathogenesis critically depends on the choice of the reference tissue. Most previous studies of ESFT expression profiles used sarcomas or the mean of different normal tissues for reference [5]–[7]. Guided by the hypothesis that upon malignant conversion of the still enigmatic ESFT progenitor cell most of the expression changes should be related to the presence of EWS-FLI1, and therefore genes that are repressed/activated after EWS-FLI1 silencing in the cell lines should be expressed at higher/lower levels in primary ESFT than in the appropriate reference tissue, we found that MPC best fit this assumption. Using the largest panel of EWS-FLI1 knockdown cell lines reported so far and doubling the number of primary tumors in the analysis, our data support the conclusions of Tirode et al. about the possible relation of ESFT to mesenchymal progenitor cells [8]. Using MPC as reference and selecting genes that were differentially expressed (FDR<0.1, GS1) in both data sets (ΔKD *and* ΔET) only a small subset (∼6%) of genes was found not to follow the theoretical expectation.

To assess the consequences of choosing MPC as reference tissue for ESFT gene expression, we checked for genes that were defined in earlier studies as putative EWS-FLI1 targets. We found that the overlap with a recently published EWS-FLI1 signature [4] was significant, but ∼70% of the genes at a p-value cut-off of 0.1 (GS1) and ∼80% at a stringency threshold of 0.25 (GS2) were not present in this earlier data set. Conversely, ∼75% (overlap with GS1) and ∼60% (overlap with GS2) of the Hancock 2007 signature were not found in our study. This is illustrated in Figure S2 which shows the distribution of ΔKD and ΔET values for all genes from the Hancock signature. Genes that were not included in GS1 or GS2 did not show a significant p value for ΔKD or ΔET, and therefore they are located around the intersection of the zero-lines in the Figure. Still, however, most genes that were found to be up-regulated by EWS-FLI1 in the Hancock meta-analysis are found in the upper left quadrant of Figure S2A, while most genes that were found to be down-regulated by EWS-FLI1 in the previous study are found in the lower right quadrant of Figure S2B. Thus, even though not significant in our analysis, the majority of the Hancock signature genes tend to conform to the underlying hypothesis of our study of EWS-FLI1 being the major driver of aberrant gene expression in ESFT, despite the usage of different reference tissues. A DAVID analysis on the Hancock signature genes revealed that they annotate preferentially to identical functional categories as identified in our study (not shown). Consequently, the two EWS-FLI1 signatures gradually differ with respect to their significance levels but not in their functional assignments. Therefore, in Table S1 ΔKD and ΔET values are provided for all genes and any gene can be assessed in relation to the rank order of all other genes that were studied here. Ranking the genes by their combined log2 fold change (ΔSUM = abs(ΔKD)+abs(ΔET)) identified *PRKCB1*, *NR0B1*, *NKX2-2*, *BCL11B*, *KMO*, *OPN3*, *PPP1R1A*, *PKP1*, *DAPK1*, *CCK*, *TRPM4* (EWS-FLI1 up-regulated) and *RND3*, *PTX3*, *LOX*, *TFPI2*, *ADM*, *TAGLN*, *DAB2*, *SRPX*, *GADD45B* (EWS-FLI1 repressed) as the 20 top EWS-FLI1 regulated genes. However, only 11 of these are found in the Hancock signature. On the other hand most of these genes are classified as high ranking in the Tirode data set [8] (Table S2), which is expected as the present study was similarly designed, however with more data from primary tumors and cell lines. Furthermore, some genes that were reported as putative “bona fide” targets of EWS-FLI1 in ESFT are not found in the highest ranking gene set in our study. From a list of earlier identified EWS-FLI1 target genes which were also biochemically studied in some detail (*CAV1* [25], *DKK1* [26], [27], *ID2* [28], *IGF1* [29], *IGFBP3* [30], *MAPT* [20], *NKX2-2* [31], [32], *NR0B1* [15], [33], *PDGFC* [34], *PLD2* [35], *PTPN13* [36], *STEAP1* [31], *TGFBR2* [37], *TERT* [38], *UPP1* [39]), 33% (*CAV1*, *ID2*, *IGF1*, *PLD2* and *TERT*) were neither found in GS1 nor in GS2. These genes rank at positions 1322, 1590, 847, 4437 and 5482 of the whole gene list, respectively (Table S1, sheet1 ranked by ΔSUM).

Of note, most of the above so far in-depth studied genes, regardless whether EWS-FLI1 up- or down-regulated, are mainly annotated to be involved in signaling, morphogenesis, development and other more specialized functions. When compared to the molecular function map developed in this study (Figure 3) these annotations overlap with only a subset of the molecular functions of EWS-FLI1 deregulated genes. In contrast, the majority of EWS-FLI1 activated genes relate to cell cycle and functions that are needed for the cell to proliferate at a high rate (“mitochondrion”, “rRNA processing”, “biosynthetic process”, etc). Furthermore, the most significant protein interactions in the data suggested that DNA-repair complexes are activated by EWS-FLI1. The almost exclusive enrichment of E2F sites in up-regulated genes might hint to a special importance of E2F transcription factors in EWS-FLI1 mediated proliferation control. On the other hand, genes that are known to function in cell differentiation, morphogenesis and the formation of differentiated tissues were generally down-regulated by EWS-FLI1, consistent with the poorly differentiated ESFT phenotype. Thus it appears that EWS-FLI1 combines two key functions in oncogenic transformation, proliferation stimulation and suppression of differentiation, in one protein, but by distinct mechanisms.

Based on our results, one would expect that introduction of EWS-FLI1 into MPC or mesenchymal cell lines should also result in a cell cycle signature. While this was the case for the human fetal fibroblast cell line IMR90 [40], DAVID annotation of signature genes in human MPC cultures with ectopically expressed EWS-FLI1 did not highlight enrichment of cell cycle or related functions consistent with the reported lack of any change in the proliferation rate of these cells [9]. We can only speculate about the possible reasons for this apparent discrepancy to the EWS-FLI1 knock-down models and ESFT tumors. It may be related to the in-vitro culture conditions for the expansion of MPC.

Another difference of EWS-FLI1 up- and down-regulated classes of genes in ESFT was revealed in the pGSEA analysis of the MSigDB signatures. MSigDB gene collections overlapping with EWS-FLI1 up-regulated genes showed relatively strong clustering due to the similarity of the respective gene sets, while down-regulated genes showed only little overlap. Thus MSigDB gene collections enriched for EWS-FLI1 up-regulated genes, despite being derived from different sources, seem to belong to a functional class that is characterized by a relatively invariant gene set, while MSigDB signatures enriched for EWS-FLI1 down-regulated targets are more variable in gene composition. The analysis of the motif gene sets (C3) shows this pattern most clearly: While for the EWS-FLI1 up-regulated genes only enrichment of E2F, NRF1, NFY motifs was found, a wide variety of DNA sequence motifs was identified in EWS-FLI1 down-regulated genes. This finding suggests a more complex transcription-regulatory network acting upstream of the EWS-FLI1 down-regulated than of EWS-FLI1 up-regulated genes, and raises the question, in how far and in which way EWS-FLI1 directly controls gene regulation by directly binding to its targets. Previous in-vitro studies identified EWS-FLI1 as an activating transcription factor [41], [42]. Thus, one would expect more direct EWS-FLI1 targets among activated than among repressed genes, and our data suggest that these predominantly map to cell cycle and proliferation control. In this respect, the association of EWS-FLI1 activated genes with E2F motifs in C3 may be of interest. Surprisingly, only 5.5% of the EWS-FLI1 up-regulated genes and 2.9% of the down-regulated genes in GS1 are represented among directly bound genes in a recently published EWS-FLI1 ChIP-on chip study [15]. Whether this low frequency of gene discovery reflects either a tight spectrum of genes in the top layer of the EWS-FLI1 target gene hierarchy or may be due to methodological limitations, or suggest a transcription-independent mechanism in EWS-FLI1 mediated gene regulation remains to be established in future studies.

The pGSEA analysis of the MSigDB collection C1 revealed a positional bias for EWS-FLI1 regulated genes with respect to chromosomal localizations. Increased expression in ESFT versus MPC was generally found to be enriched on chromosomes 8, 14, 17, 19, and 22, especially at the terminal regions, and to a lesser extent on chromosomes 12, 13 and 20. Two chromosomal regions deserve special attention: 8q24, the region comprising the MYC locus, was found over-expressed, and 16q12–13 showed significant under-expression. Both chromosomal regions are frequently involved in numerical aberrations in ESFT. Trisomy 8 occurs in about 50% of cases, and losses of 16q are frequently observed in ESFT due to unbalanced rearrangements with chromosome 1, and are associated with poor outcome [43]. While it cannot be excluded that the observed enrichment for chromosome 8 encoded genes is a consequence of a high content of chromosome 8 amplified tumors in the investigated ESFT series, it should be noted that chromosome 8 was also enriched in a previous EWS-FLI1 ChIP-screening study [44], suggesting that increased expression of this genomic region might be driven by EWS-FLI1 and is amplified by chromosome duplication. Also, under-expression of 16q12–13 cannot easily be explained by chromosomal deletion, since several of the genes under-expressed in ESFT were found to be significantly induced upon knockdown of EWS-FLI1 and were therefore found in GS1 and GS2 (i.e. *MMP2*, *AKTIP*, *CYLD* ), while others remained unchanged (i.e. *ZNF423*, *N4BP1*, *ITFG1*). Overall the lowest expression values on chromosome 16 were found in 16q13 (Figure S2) which was therefore identified as significant in the pGSEA analysis. Here, functional annotation revealed enrichment of “chelation/metal ion”, which is due to the fact that all metallothionein genes reside in this region. Since these proteins have previously been implicated in cancer [45], [46], their suppression in ESFT may deserve in-depth functional studies in the future. Two further genes from this region, *HERPUD1* and *ARL2BP*, were also found suppressed, while *NUP93*, which separates them from the metallothionein cluster, showed slightly higher expression in ESFT than in MPC.

In summary, our data are consistent with a mesenchymal progenitor cell origin of ESFT and suggest a dual role of EWS-FLI1 in ESFT pathogenesis: the activation of cell cycle and proliferation promoting genes and the repression of differentiation. Our results may guide target selection for future molecular therapeutic intervention strategies in ESFT.

## Materials and Methods

### Cell lines

All ESFT cell lines used in this study have previously been described [47], [48]. Cell lines WE68, SK-N-MC, and TC252 were kindly supplied by F. Van Valen (Dept. of Pediatrics, University of Muenster, Germany), J. Biedler (Memorial Sloan-Kettering Cancer Center, New York, USA), and T. Triche (Dept. of Pathology, Children's Hospital, Los Angeles, USA), respectively. The STA-ET-1 and STA-ET-7.2 cell lines were established at the Children's Cancer Research Institute (Vienna, Austria).

### RNA interference

Cells were transfected with LipofectAMINE Plus reagent (Invitrogen, Groningen, The Netherlands) and subjected to puromycin selection (1 µg/mL) the next day. On day 4 after transfection, puromycin selected cells were processed for RNA and protein extraction. SiRNAs against the fusion regions of type 1 (EF30) in TC252, WE68, SK-N-MC, STA-ET-1, and type 2 (EF22) EWS-FLI1 in STA-ET-7.2 were expressed as small hairpin (sh) RNAs from pSUPER-based retroviral expression constructs as previously described [12]. For negative control, pSTNeg (Ambion, Applied Biosystems, Brunn am Gebirge, Austria) encoding a scrambled shRNA with no significant similarity to human sequences was used.

### Gene expression analysis by microarray technology

Changes in gene expression profiles upon knockdown of EWS-FLI1 were followed on Affymetrix HG-U133A arrays (Affymetrix, Inc., Santa Clara, CA). cRNA target synthesis and GeneChip® processing were performed according to standard protocols (Affymetrix, Inc., Santa Clara, CA). All further analyses were performed in R statistical environment using Bioconductor packages [49]. Microarray data from the knockdown analysis was submitted to GEO – accession number: GSE14543.

Affymetrix CEL files for i) five ESFT cell lines (TC252, SKNMC, STA-ET-7.2, STA-ET-1, WE68, knockdown and control), ii) expression experiments from 3 different datasets of molecularly confirmed primary ESFT (Henderson et al. 2005: n = 5, Schaefer et al. 2008: n = 27, Tirode et al. 2007: n = 27, [6], [7], [8] (ArrayExpress: E-MEXP-353), (ArrayExpress: E-MEXP-1142), (GEO: GSE7007). and iii) the “Novartis gene expression atlas” which comprises 79 tissues [13] (GEO: GSE1133) and iv) mesenchymal progenitor cell data [8 (GEO: GSE7007),14 (ArrayExpress. E-MEXP-167)] were normalized together using the gcrma logarithm [50]. For further analysis probesets (ps) were called present or absent using the Bioconductor package “panp”, and ps were excluded which showed very low expression across all arrays (P>0.05). Subsequently ps were excluded that did not pass a non-stringent variability cut-off across the 10 EWS-FLI1 cell line arrays (interquantile range>0.2). Thereby ps were excluded that did not show variation in the EWS-FLI1 knock-down compared to control experiments. Finally, for each gene one ps was selected for further analysis by the criterion of maximizing the expression variation across arrays and thereby the most informative ps was chosen for each gene. This procedure yielded a final number of 7034 ps that were used for all further analysis. A list of used probe sets can be found in Table S1.

Hypothesis free clustering of the 1000 most variable genes in the data set shows that, despite being from three different sources, ESFT form a group distinct from all other tissues that were analyzed (Figure S1) and within this cluster ESFT from the three sources are intermixed. This demonstrates that an often observed “batch effect” [51] does not distort the similarity pattern in this data set. Furthermore the experiments from the five ESFT cell lines are placed next to the primary tumors in the resulting dendrogram further corroborating the biologically meaningful clustering of samples.

Differentially expressed genes were determined using a moderated t-test in the R package “limma” [52] and a non-parametric method provided in the R package “RankProd” [53]. All P values were corrected for multiple testing using the “Benjamini-Hochberg” correction method. For the determination of significantly changing genes in the EWS-FLI1 knock-down compared to control experiments first ratios for each gene were calculated between the two conditions for each cell line separately, thus yielding five biological replicates of relative expression for each gene. Then for each gene significance was determined using the equivalent of a one-sample t-test in limma and RankProd. Using the same false discovery rate, the resulting gene lists from both methods largely overlapped and significant genes from both methods were combined. To identify differently expressed genes between ESFT and a reference, a two-sample moderated t-test was performed in limma. ESFT samples from the three above mentioned sources were averaged. For MPC, bone marrow mesenchymal progenitor cell data from Tirode et al [8] and CD31-minus samples from Boquest et al [14] were combined and for the tissues in the Novartis gene atlas the two replicates for each tissue were averaged.

To select genes from gene sets differentially expressed in EWS-FLI1 knockdown experiments (deltaKD) and in primary ESFT (deltaET) for intersection, we deliberately chose two cut-offs of different stringencies that would allow to maximize the number of genes for functional annotation in GS1 and GS2 at a reasonably significant p-value. Choosing thresholds (FDR) of 0.1 and 0.25 fulfilled this requirement by producing a large enough data set for functional annotation, while the random expectation for a gene being present in both deltaKD and deltaET data sets at these thresholds is only 0.01 and 0.025, respectively.

### Functional annotation

The “Database for Annotation, Visualization and Integrated Discovery” (DAVID) was used to annotate the 344 up- and 237 downregulated genes of GS1. The “Functional Annotation Tool” in the online version of DAVID was run (http://david.abcc.ncifcrf.gov/) using the default parameters and additionally protein interaction data from the “BIND”, “DIP”, “MINT” and “NCICB_CAPATHWAY” as annotation databases. The “Functional Annotation Clustering” tool was used to combine significant annotation terms into functional groups (FG) using default parameters. The output from this analysis can be found as supplementary Table S2, S3. The genes for each FG in these Tables are the intersection between the annotation terms from the data bases and the genes present in GS1.

### Multidimensional scaling (MDS) and hierarchical clustering (HC)

To visualize the relationships of the functional groups from the functional annotation analysis (Table S2, S3) first similarity between all groups was measured by the number of their shared genes (gDist). For each pair of FGs gDist was calculated as gDist = *N*(A)∩*N*(B)/min(*N*(A), *N*(B)), where *N*(A) denotes the number of genes in FG (A). Therefore, a similarity of 1 indicates that the smaller gene set is a subset of the larger gene set. All genes of an FG were used for the calculation. The matrix of pair wise gDist values (as dissimilarities: 1-gDist) for the 25 most significant FGs from DAVID was used as input for MDS and HC. For MDS the R function “sammon” from the R package MASS was used with default parameters. In brief, the procedure, which is a form of non-metric multidimensional scaling, iteratively attempts to place the samples (FGs) in n-dimensional space to reflect their similarity as measured by gDist, while minimizing the overall “Stress” statistic. This statistic is based on the sum of squared differences between the input distances and those of the configuration. Therefore, it converges at an optimal solution for visualizing similarity/dissimilarity of all samples in n-dimensional space. Here a solution for two dimensions was sought. The coordinates of the FGs on the first two axes (dimensions) of the MDS are shown in Figure 3. Additionally, circles were drawn around the coordinates of FGs were the size of the circles indicates the relative size (number of genes) of the FGs. Therefore FGs with a distance of 0 can be visualized.

For HC and the heatmap in Figure 3 the R function “hclust” was used in combination with the “heatmap.2” function using the “average linkage” algorithm. For the labels of the FGs in Figure 3, the most significant annotation term within each FG was chosen (see Table S2, S3).

### pGSEA analysis

The gene set collections C1, C2, C3 of the MSigDB were downloaded from the Broad institute (http://www.broad.mit.edu/gsea/msigdb/) and loaded into R statistical environment. The PGSEA package [17] was used to calculate z-scores for all gene sets in MgSigDB using the mean log2-foldchange for ΔKD and ΔET. All significant MSigDB gene sets along with gene IDs can be found in Table S4. For the identification of the ten most significant gene sets in C2–C3 the sum of the absolute values of the two z-scores (for ΔKD or ΔET) for each gene set was calculated and the gene IDs for highest scoring gene sets were extracted. Based on the gene IDs of these gene sets gDist was calculated for all pair wise combinations of up- and downregulated gene sets separately. The resulting two matrices were used as input for HC as described above. The two combined gene lists for up- and down regulated genes were also used as input for functional annotation with DAVID as described above.

## Supporting Information

Figure S1 Heatmap of the 1,000 most variable genes. Variability of genes across samples was measured as standard deviation. Hierarchical clustering was performed using “euclidian distance” and “complete linkage.” The color bar on top of the heatmap indicates the sample affiliations: Red - primary ESFT, orange - ESFT cell lines (RNAi control), green - ESFT cell lines (RNAi knockdown), blue - tissues from the Novartis gene expression atlas. The color ramp white-yellow-orange-red is used to display the log2 expression values (after gcrma normalization) of each gene in each sample. Primary ESFT form a cluster independent of the data source.
(6.85 MB TIF)

Figure S2 Comparison with the Hancock et al 2008 EWS-FLI1 signature. Plot of relative gene expression values of the EWS-FLI1 knockdown compared to control (deltaKD, x-axis) and ESFT compared to reference (deltaET, y-axis) for genes that were found significant in Hancock et al 2008. (A) Genes found to be up-regulated in the Hancock data set. (B) Genes found to be down-regulated in the Hancock data set. Red/dark blue dots denote genes that were found in GS1 in our study; Pink/light blue dots denote genes that were found in GS2 in our study; Grey dots denote genes that are neither in GS1 nor in GS2.
(0.32 MB TIF)

Table S1 Differential expression - genelists. List of differential expression values and annotation (ΔKD-ΔET) for all genes/probesets and for subsets meeting significance level cutoffs (GS1, GS2).
(2.18 MB XLS)

Table S2 DAVID Analysis - up. Output from DAVID analysis for EWS-FLI1 upregulated genes in GS1.
(0.30 MB XLS)

Table S3 DAVID Analysis - down. Output from DAVID analysis for EWS-FLI1 downregulated genes in GS1.
(0.22 MB XLS)

Table S4 pGSEA Analysis. Output from pGSEA analysis.
(0.16 MB XLS)
