# Supplementary figures and images for: A Molecular Function Map of Ewing's Sarcoma
Source: PLoS One. 2009 Apr 30;4(4):e5415. doi: 10.1371/journal.pone.0005415 (PMC2671847; doi:10.1371/journal.pone.0005415)

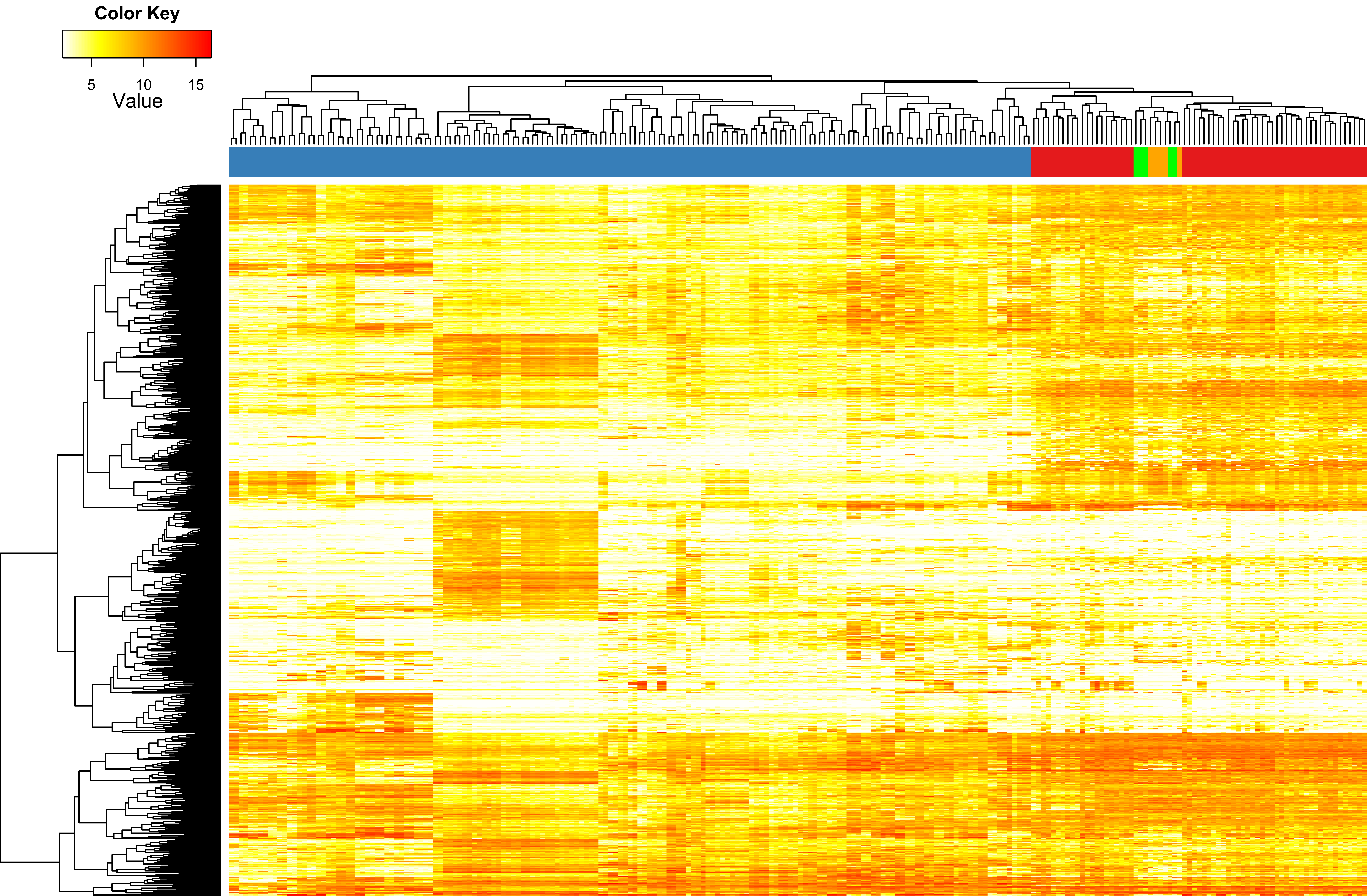

Supplement: Figure S1 — Heatmap of the 1,000 most variable genes. Variability of genes across samples was measured as standard deviation. Hierarchical clustering was performed using “euclidian distance” and “complete linkage.” The color bar on top of the heatmap indicates the sample affiliations: Red - primary ESFT, orange - ESFT cell lines (RNAi control), green - ESFT cell lines (RNAi knockdown), blue - tissues from the Novartis gene expression atlas. The color ramp white-yellow-orange-red is used to display the log2 expression values (after gcrma normalization) of each gene in each sample. Primary ESFT form a cluster independent of the data source. (6.85 MB TIF) [file pone.0005415.s001.tif]

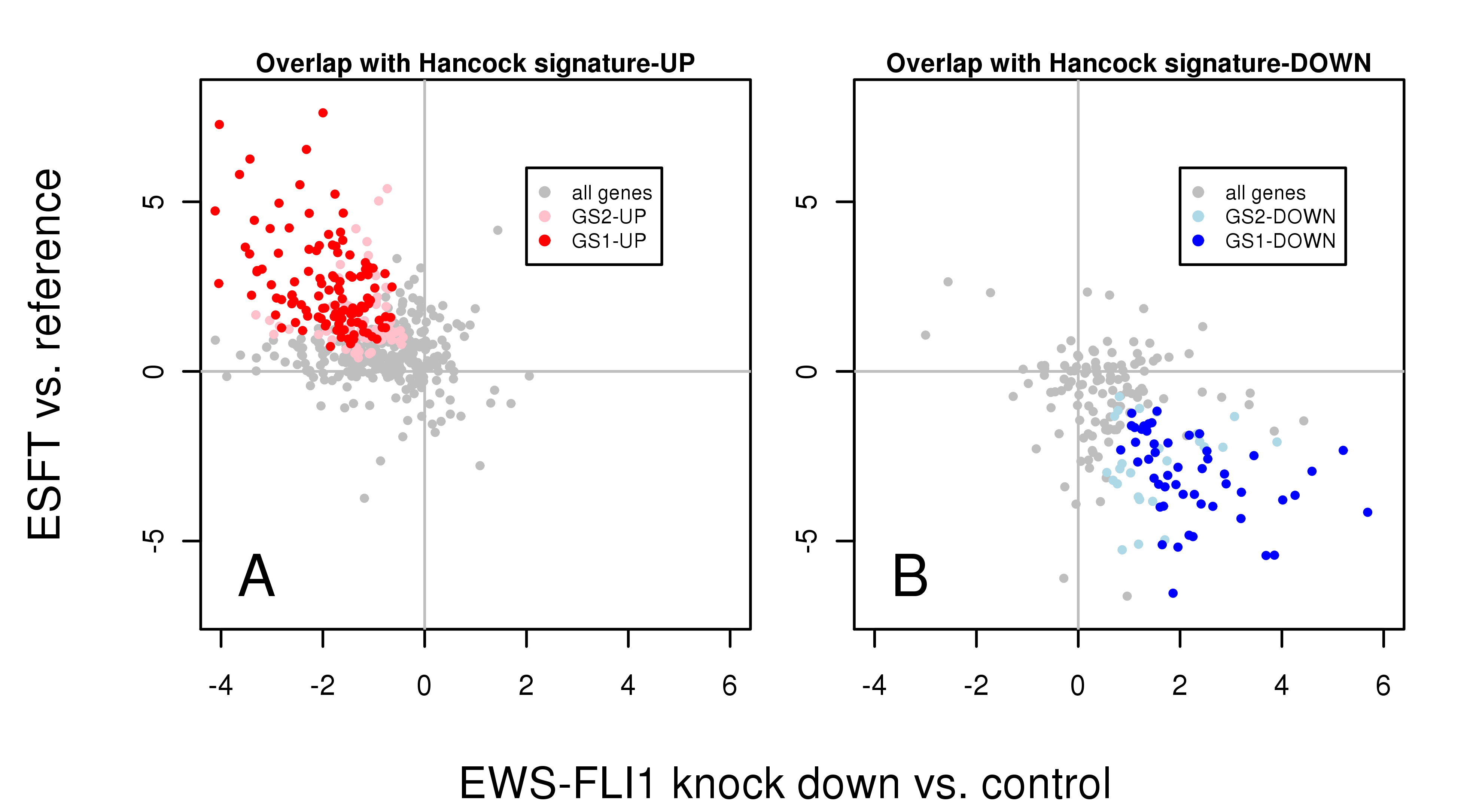

Supplement: Figure S2 — Comparison with the Hancock et al 2008 EWS-FLI1 signature. Plot of relative gene expression values of the EWS-FLI1 knockdown compared to control (deltaKD, x-axis) and ESFT compared to reference (deltaET, y-axis) for genes that were found significant in Hancock et al 2008. (A) Genes found to be up-regulated in the Hancock data set. (B) Genes found to be down-regulated in the Hancock data set. Red/dark blue dots denote genes that were found in GS1 in our study; Pink/light blue dots denote genes that were found in GS2 in our study; Grey dots denote genes that are neither in GS1 nor in GS2. (0.32 MB TIF) [file pone.0005415.s002.tif]
